# Discordant spatiotemporal dynamics of functional and phylogenetic diversity of rotiferan communities exposed to aquaculture effluent

**DOI:** 10.1002/ece3.10503

**Published:** 2023-09-05

**Authors:** Flórián Tóth, Katalin Zsuga, Éva Kerepeczki, Balázs Kovács, Tibor Magura, László Körmöczi, Gábor L. Lövei

**Affiliations:** ^1^ Department of Hydrobiology, Research Centre for Aquaculture and Fisheries, Institute of Aquaculture and Environmental Safety Hungarian University of Agriculture and Life Sciences Szarvas Hungary; ^2^ Department of Ecology, Faculty of Science and Informatics University of Szeged Szeged Hungary; ^3^ Department of Agroecology Flakkebjerg Research Center, Aarhus University Slagelse Denmark; ^4^ Agrint Kft Gödöllő Hungary; ^5^ Department of Molecular Ecology, Institute of Aquaculture and Environmental Safety Hungarian University of Agriculture and Life Sciences Gödöllő Hungary; ^6^ ELKH‐DE Anthropocene Ecology Research Group University of Debrecen Debrecen Hungary; ^7^ Department of Ecology, Faculty of Science and Technology University of Debrecen Debrecen Hungary

**Keywords:** biodiversity, community assembly, limnology, Rao's diversity, zooplankton

## Abstract

The growth of the human population brought about the global intensification of aquacultural production, and aquaculture became the fastest growing animal husbandry sector. Effluent from aquaculture is an anthropogenic environmental burden, containing organic matter, nutrients and suspended solids that affect water quality especially in the water bodies of high biodiversity and conservation value. Water quality assessment often relies on bioindicators, analysing changes in taxonomic diversity of various freshwater organismal groups. Stepping beyond taxon diversity, we used functional and phylogenetic diversities of rotifers to identify factors affecting their community organization in response to an aquaculture effluent gradient in the largest oxbow lake in the Carpathian Basin, Hungary. Sampling was carried out three times per season at five points along a 3.5 km section of the oxbow lake, including the point of effluent inflow. We used eight traits to evaluate functional diversity: body size, trophi type, feeding mode, protection type, body wall type, corona type, habitat preference and tolerance level. Functional and phylogenetic distances among the 24 species identified indicated trait conservatism. Rotiferan diversity increased with increasing distance from the point of influx in spring and summer. Among the factors affecting community organization in spring and summer, we find examples of environmental filtering, while in autumn the role of biotic interaction is more frequent. Under nutrient‐rich conditions in spring and summer, organisms belonging to the same functional group were dominant, whereas under oligotrophic conditions, more diverse but less abundant groups were present. Considering functional and phylogenetic traits allowed us to identify organising forces of rotifer communities in the largest oxbow lake of the Hungarian Lowland.

## Graphical abstract

Effluent from aquaculture is an anthropogenic environmental burden, containing organic matter, nutrients and suspended solids that affect water quality in water bodies of high biodiversity and conservation value. Stepping beyond taxon diversity, we used functional and phylogenetic diversities of rotifera to identify factors affecting their community organization in response to an aquaculture effluent gradient in the largest oxbow lake in the Carpathian Basin. Rotiferan diversity increased with increasing distance from the point of influx. In spring, rotiferan communities were organised by environmental filtering, whereas during autumn, biotic interactions became influential factors. Under nutrient‐rich conditions in spring and summer, organisms belonging to the same functional group were dominant, whereas under oligotrophic conditions, more diverse but less abundant groups were present.

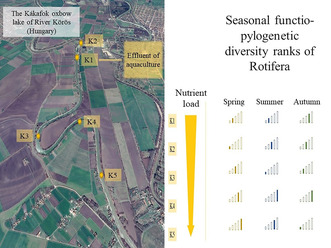

## 1 INTRODUCTION

Currently, humankind collectively uses more than half of the world's available freshwater (FAO, 2022). Water is an essential resource for food production, not only for irrigation but also for aquaculture, which is now a more important global source of protein than fisheries (FAO, 2018). Global human population pressures increase the demand for aquaculture products, posing new environmental challenges to this sector. Aquaculture, currently one of the fastest growing branch of the global animal husbandry sector (FAO, 2018) has profound habitat‐modifying effects. With the intensification of aquaculture, both feed consumption and the amount of waste increases, causing increased effluent loads of organic matter and nutrients (Edwards, 2015), with potentially negative environmental impacts (Naylor et al., 2000). Phosphorus, nitrogen and organic matter from unconsumed feed and metabolites are the most common problems, causing oxygen deficiency and eutrophication (Gál et al., 2016). Understanding the impacts of aquacultural production practices on various freshwater communities has practical as well as theoretical ecological importance.

The nutrient load from aquaculture is combined with other pressures on all kinds of water bodies but especially on wetlands, whose area is decreasing worldwide (Čížková et al., 2013). Wetlands support valuable biodiversity, provided their water quality does not deteriorate. The worldwide assault on rivers accelerated in the 19th century, when many European rivers became ‘regulated’. By straightening the formerly meandering rivers, many natural wetlands disappeared, but a significant number of oxbow lakes were created. There are 287 oxbow lakes in Hungary alone (Pálfai, 2001).

These oxbow lakes in the Carpathian Basin became significant nature conservation areas (Ortmann‐Ajkai, 2019), improving the stability of floodplain ecological corridors (Kerényi & Szabó, 2007). Oxbow lakes are also important areas for local communities, but excessive nutrient loading poses a significant threat to them (Carp, 1972). The EU Water Directive prescribes regular water quality monitoring of such water bodies (Chave, 2001).

One of the most common biological approaches to understanding the various effects of human‐induced environmental stress on water quality is to examine and record changes in the diversity of various freshwater taxa. Several measures of diversity (including taxonomic, genetic, phylogenetic and functional diversities) show a rapid decrease in response to anthropogenic effects (Naeem et al., 2012).

Using taxonomic diversity as a response variable to indicate various kinds of environmental ‘stress’ has the inherent problem of ignoring species roles: ‘a species is a species’, and the importance of any given species in the ecosystem is irrelevant in such an evaluation. However, ecological functioning crucially depends on what the species present in a habitat actually do (Cadotte et al., 2011). This raises the possibility that analysing diversity at the functional level is more meaningful when we aim to evaluate the impact of various environmental stressors. However, while the theoretical basis of species diversity, namely the species concept, is well established, the same cannot be said about functional diversity (Moretti et al., 2017). Nonetheless, over the last decade, the focus of diversity‐based analyses shifted from taxonomic to trait‐based approaches (Obertegger & Wallace, 2023). Using functional diversity constrains the possible parameter space (there are more species than species traits), but enables generalizations across communities and ecosystems that are needed to investigate the general consequences of the various changes—often due to anthropogenic stressors—for ecosystem processes (Hortal et al., 2015; Kunstler et al., 2016; McGill et al., 2006).

Differences (or similarities) between species traits are crucial for community structure, as organising mechanisms affect similarities and differences between constituent organisms (Cadotte et al., 2013). To select traits, we can regard two features: (1) Response to a change in an environmental factor or to an interaction with another organism. (2) Effect of the species on an interaction or on an ecosystem function. Both can occur at the same time (Lavorel et al., 2013; Naeem & Wright, 2003). Additionally, given that species belonging to a similar functional group may utilize the same resources, and related species have common morphological and ecological characteristics, functional and phylogenetic information can be used to quantify such differences (Webb et al., 2002). Phylogenetic relatedness will influence the differences in traits among species (closely related species will have more similar traits); however, phylogenetically distant species can also share the same function and/or habitat (Mayfield & Levine, 2010). Thus, it is prudent to consider phylogenetic relationships together with functional diversity.

Rotifers are microscopic invertebrates that play an essential role in the food web of aquatic habitats and in transporting energy from lower to higher trophic levels (Wallace et al., 2006). Rotifers are among the fastest reproducing organisms, and due to these features, they are able to respond quickly to environmental changes (Allan, 1976). Rotifers have been used to characterize eutrophication (Berzins, 1949; Jarnefelt, 1952; Lillieroth, 1950; Pejler, 1957, 1965; Thunmark, 1945), saprobity (Gulyás, 1983; Sládeček, 1983) and to develop trophic state indices (Ejsmont‐Karabin, 2012; Ochocka & Pasztaleniec, 2016).

Community organization examined by functional groups for rotiferan communities can be instructive. Oh et al. (2017) showed that the species characteristic of different trophic states reacted differently to the degree of eutrophication, indicating that each group may be affected differently by combinations of water quality variables. Obertegger and Manca (2011) using functional instead of traditional taxonomic indices showed a dramatic shift from a predator dominance to a microphagous one, with a subsequent back‐shift after several years. Rotiferan communities of an Estonian shallow eutrophic lake have small predators as the most important functional group in winter, while suckers are absent and fine particle sedimentators have a marginal role (Virro et al., 2009). In Lake Tovel in Italy, rotiferan trait convergence dominates in the upper layer driven by environmental filtering and trait divergence in lower layers driven by species interactions (Obertegger & Flaim, 2015). These studies, while reporting informative changes in rotifer community composition, did not consider phylogenetic diversity.

The Kákafok oxbow lake receives aquaculture effluent from a nearby fish farm, providing a substantial, point‐source nutrient subsidy. A previous study identified the presence of 26 rotifer species, with the highest taxonomic diversity at the point of nutrient inflow in summer and autumn but not during spring (Tóth et al., 2020). Evaluating the combined functional and phylogenetic diversity, we aimed to determine the impact of this nutrient subsidy on the spatiotemporal organization of rotifer communities. Under nutrient‐rich conditions, a community with a smaller number of species but high abundance usually develops, while under oligotrophic conditions, there are more species, each with low dominance (Gliwicz, 1969). The consequences of such conditions on functional and phylogenetic diversity have rarely been studied. We hypothesized that the diversity based on functional and phylogenetic approaches increased from the point of inflow of the nutrient‐rich effluent in each season, because the concentration of nutrients decreases as a result of dilution due to distance.

## 2 METHODS

### 2.1 Study area and sampling methods

The Kákafok oxbow lake, stretching between the south Hungarian settlements of Szarvas and Békésszentandrás (46°51′14.9″ N, 20°30′44.6″ E) is 29 km long and was separated from the River Körös during the 19th century river regulations. This is the biggest flood‐protected oxbow lake of the River Tisza watershed and also the fifth largest stagnant water body in Hungary, with an average depth of 2.2 m and an area of 207 ha; it contains about 4.5 million m^3^ of water (Pálfai, 2001). It is a semipaleopotamonic water body, with one end very near to the parent river, from where a pumping station pumps water into the oxbow in early spring (Józsa et al., 2017). In late autumn, the level in the oxbow is reduced by retro‐pumping water into the parent river. There is no significant water in‐ or out‐flow between these periods, and thus the water body can be considered stagnant.

**FIGURE 1:**
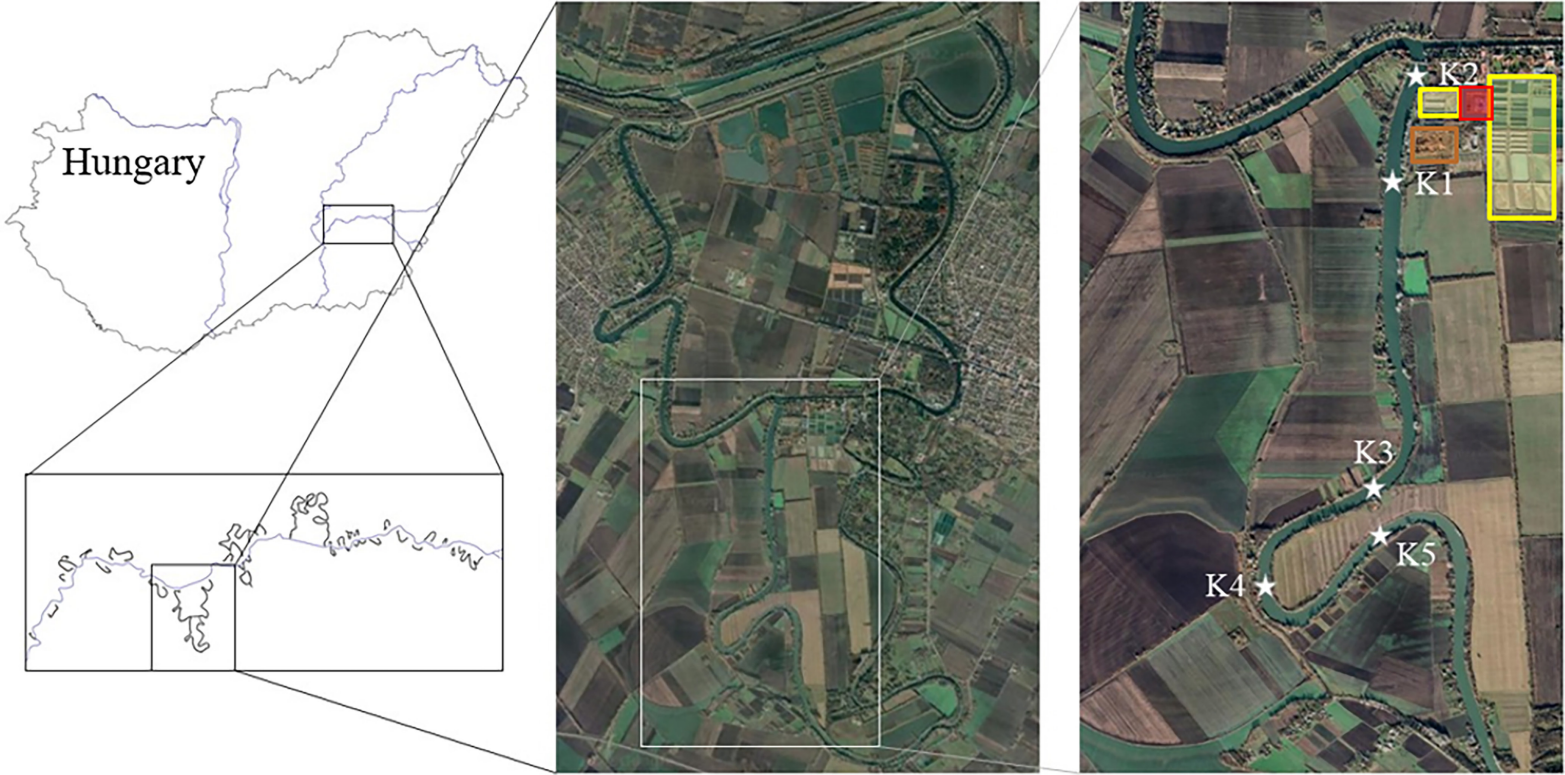
Positions of the sampling points (K1–K5) of the Kákafok oxbow lake in Hungary. Yellow tetragons: the experimental fishpond system of MATE HAKI, red tetragon: the African catfish (*Clarias gariepinus*) farm, orange tetragon: artificial wetland water treatment system. The bridge above point K2 blocked further sampling points in that direction. (*Source*: Google Earth).

Five sampling points (K1–K5) were selected along a 3.5 km long section of the oxbow lake. The first of these is the introduction point for effluent water from an intensive African catfish (*Clarias gariepinus*) warm‐water farm and the experimental fish ponds of the Research Centre for Aquaculture and Fisheries—Hungarian University of Agriculture and Life Sciences. The amount of the catfish farm effluent was about 330,500 m^3^ year^−1^ (Tóth et al., 2018). The other four sampling sites were located at different distances from this point. There were no other known nutrient loads at this section of the oxbow lake. Thus, at point K1, the nutrient‐rich effluent water entered the oxbow lake after passing through an artificial wetland water treatment system so that there was no thermal pollution. The sampling points were selected based on the characteristics of the oxbow. Accordingly, the additional points were located at 500 m (K2), 2500 m (K3), 3000 m (K4) and 3500 m (K5) from the point of effluent. The location of sampling points was influenced by an intensive fish survey along an ca. 2 km long section, so no sampling points could be located there (Figure 1).

**FIGURE 2:**
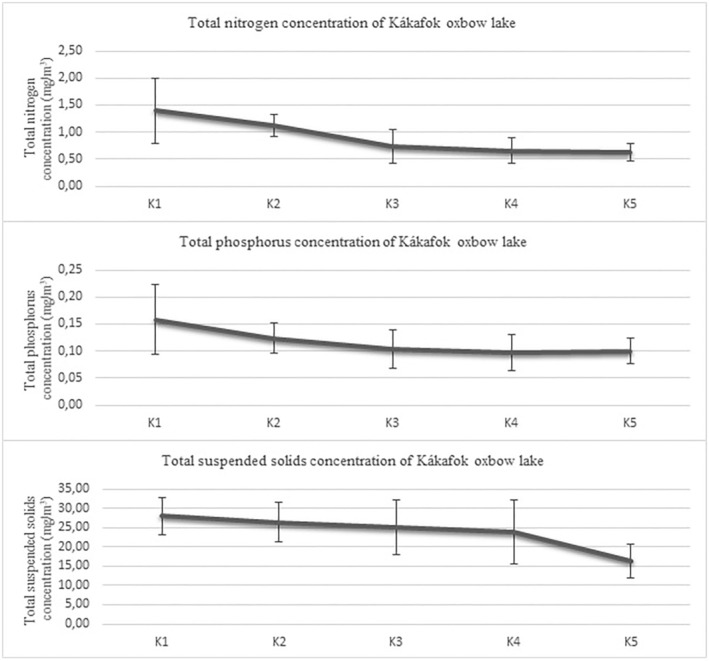
Mean concentration of total nitrogen, total phosphorous and total suspended solids of Kákafok oxbow lake.

With increasing distances from the point of inflow, the nutrient content decreased. During the study year, the total nitrogen (TN) content at point K1 varied between 0.195 and 4.000 mg/m^3^, the total phosphorous (TP) from 0.053 to 0.468 mg/L and the total suspended solids (TSS) from 13.14 to 41.10 mg/L. These values at point K5 were: TN: 0.275–1.140 mg/L, TP: 0.062–0.210 mg/L and TSS: 7.1–28.6 mg/L (Tóth et al., 2017; Table S1; Figure 2).

Rotifers were sampled three times during each season: in spring (20 April, 10 May and 1 June), summer (29 June, 21 July and 10 August) and autumn (30 August, 21 September and 24 October) of 2016. Each time, 50 L of surface water at each point was filtered through a 50 μm mesh plankton net, concentrated to 100 mL, preserved by the addition of formaldehyde (4% final concentration) and stored at 4°C until identification. Counting was performed with a 5 mL counting chamber using a microscope (40–125× magnification). For species identification, we used standard keys (Bancsi, 1986, 1988).

### 2.2 Functional and phylogenetic diversity

We used eight ecological and morphological traits in the analysis of the functional diversity of rotiferan species (Table S2).

#### 2.2.1 Body size

This was characterized by the geometric mean of the size range found in identification keys (Bancsi, 1988). The adjustment of an organism's metabolic rate to body size is generally valid (Brown et al., 2007; Gillooly et al., 2001), so body size indirectly affects subsistence.

#### 2.2.2 Trophi type

The trophi are the pharyngeal apparatus of rotifers, which is a masticatory apparatus consisting of hard, sclerotized, and articulated jaws and muscles organized in specific spatial arrangement (Sørensen, 2002; Wallace et al., 2006). The shape of the trophi provides essential information about feeding behaviour and habitat preference. Trophi shape classes were malleate, virgate, ramate, incudate, malleoramate or forcipate (Bancsi, 1988).

#### 2.2.3 Feeding type

The type of food suitable for consumption is determined by the type of trophi and the mode of feeding (Obertegger et al., 2011; Salt et al., 1978). These factors are related, but they focus on different aspects of food collection: while the feeding mode is related to the food gathering process, the trophi type can be linked to feeding strategies (Obertegger et al., 2011). Following Gilbert (2022), we used four categories: microphagous, polyphagous, macrophagous algivores and macrophagous predators. Six species belonged to genera not included in Gilbert (2022). We assigned them based on our own experience.

*Physical protection from predators*—can be active or passive based on the existence of organs and appendices necessary for movement. Active defence, such as skipping movement, increases the chances of avoiding predators compared to the passive thanatosis (feigning death) (Roche, 1987).

#### 2.2.4 Body wall (lorica) type

We used three classes: illoricate, loricate (ridged) and loricate with spines. The lorica provide structural protection against predation (Roche, 1987). The presence of caudal spines can be considered phenotypic variability as it can be influenced by the number of predators (Gilbert & McPeek, 2013), which may limit the universality of this trait.

#### 2.2.5 Corona type

The organ at the apical end of the animal is the corona. This is a ciliated region for food gathering, perception of prey items (Salt, 1987) and locomotion (Koste, 1978; Wallace et al., 2006) and is linked to mastax and trophi structures (Kutikova, 1983). This character is named after the respective characteristic genera. We distinguished *Asplanchna*‐, *Philodina‐, Euchlanis/Brachionus‐, Notommata‐, Dichranophorus‐, Conochilus*‐, *Hexarthtra/Testudinella*‐ and *Collotheca*‐type corona types (Bancsi, 1986).

So far, this approach follows the work of Obertegger and Flaim (2015), which we expanded with the following traits.

#### 2.2.6 Habitat preference

The identified species were classified as beta‐, beta/alpha‐mesosaprobic, oligo/beta‐mesosaprobic or oligosaprobic, according to the saprobic status of the waters in which they typically can be found (Gulyás, 1983).

#### 2.2.7 Tolerance

The tolerance level of each species to organic matter was also classified on a scale of 1 (narrow) to 5 (wide), according to the number of saprobic zones the species occurs (Gulyás, 1983).).

The distance between species based on functional traits (FDist) was calculated by the Gower's distance (Gower, 1971) using the *StatMatch* software package (D'Orazio et al., 2006). Gower's distances can range from 0 to 1 based on the specifics of the method.

To determine phylogenetic distances (PDist), Cytochrome Oxidase I (COI) sequences were used based on GenBank sequences (Table S3). The species for which we could not find a sequence was replaced by a closely relateüd species. The 621 bp long sequences of the COI region were aligned and the neighbour‐joining (NJ) method was used with Kimura two‐parameter (K2P) distance model to calculate genetic divergences (Kimura, 1980). A phylogenetic tree (Figure S1) was created to provide a graphical representation of the patterns of COI divergences between species using the K2P model (Saitou & Nei, 1987) using the MegaX software (Kumar et al., 2018). The tree was constructed using bootstrap test with 3000 replicates (Felsenstein, 1985) and it is drawn to scale, with branch lengths (next to the branches) to evolutionary distances. To obtain comparable scaling with functional and phylogenetic distances the branch lengths were standardized to a range of 0–1.

The relationship between functional and phylogenetic distance matrices was examined by Mantel tests (Mantel, 1967) using the *ade4* package (Dray & Dufour, 2007) using 9999 replicates.

Because functional and phylogenetic characteristics carry complementary information about species differences, Cadotte et al. (2013) proposed their combined use. He summed it up as a functional‐phylogenetic distance (FPDist):

$$\text{FPDist}={\left(\alpha {\text{PDist}}^{\rho }+\left(1-\alpha \right)\;{\text{FDist}}^{\rho }\right)}^{1/\rho },$$

where PDist is the phylogenetic distance, FDist is the functional distance, *ρ* is an integer to provide nonlinearity, while *α* is the weighting parameter that determines the relative contributions of PDist and FDist to the combined FPDist value.

When *α* = 1, the combined index is only determined by the phylogenetic distance, while for *α* = 0, the FPDist is determined only by the functional distance. *ρ* = 2 was used in the calculations, as suggested by Cadotte et al. (2013).

Increasing the weighting parameter (*α*) in steps of 0.025, 41 levels were set between 0 and 1 (Heikkala et al., 2016). For each level, the seasonal mean pairwise functional ‐ phylogenetic distance (MFPD) was calculated. Following this, effect sizes (ES)were calculated using the observed MFPD (MFPD_observed_) of the species collected at each sampling point and the same number of species randomly selected from the regional species pool (MFPD_random_) Since the null distributions did not follow a normal distribution, we used probit‐transformed *p* values as ES as suggested by Botta‐Dukát (2018). To construct null assemblages, species were chosen randomly without replacement from the species pool, with the species richness of each site kept constant. To decrease the influence of rare species, the distance values were weighted by species abundance. ES were based on null models with 999 randomizations (Webb et al., 2002) using the *picante* package (Kembel et al., 2010). If the community is structured by random process, the mean standardized ES does not deviate from zero (Webb et al., 2002). Values in the negative range indicate environmental filtering, while positive values indicate biotic interactions as community organising forces (Pausas & Verdu, 2010; Webb et al., 2002). Confidence intervals for ES after 999 iterations were calculated using the *boot* package (Davison & Hinkley, 1997). In the next step, we determined the optimal value of the weighting parameter (*α*), where the explanation of the pattern is maximized. The strength of the relationship between the distance from the effluent influx and the standardized ES was tested step by step changing the weighting parameter (*α*) using linear models with the *lm* function. The optimal value of the weighting parameter (*α*) fell where the adjusted *R* ^2^ value of the linear model of the standardized ES and the sampling position reached its maximum (Bässler et al., 2016; Cadotte et al., 2013; Heikkala et al., 2016). Finally, the mean standardized ES were calculated using this optimal weighting parameter.

The Rao's quadratic entropy (Rao et al., 1973) was used to calculate diversity based on the combination of functional and phylogenetic distances with alpha corresponding to the maximum value of adjusted R^2^ in all seasons. This was done with the *SYNCSA* (Debastiani & Pillar, 2012) software package.

All analyses were performed in the R software environment (R Development Core Team, 2021) (R x64 4.1.2 version).

## 3 RESULTS

Functional and phylogenetic diversities were correlated (Mantel test: *R* = .2621, *p* = .026).

The alpha value with the greatest explanatory power differed in each season. The optimal values were *α* = .250 in spring, *α* = .050 in summer and *α* = .100 in autumn. In subsequent calculations, the ES values with maximum explanatory power corresponding to the season were used. In all seasons, the explanatory power of the phylogenetic component was much lower than the functional component.

**FIGURE 3:**
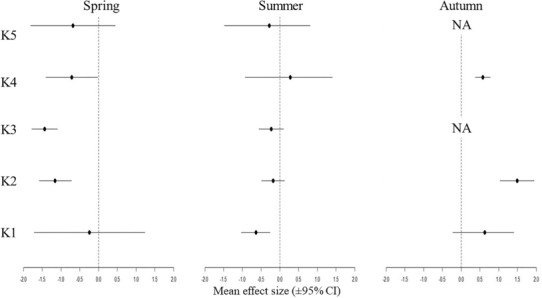
The mean effect sizes (ES) calculated with functional‐phylogenetic weighting parameter value of *α* (.25 in spring, .05 in summer and .1 in autumn) per season in the sampling points depending of the distances from the effluent point. CI means confidence interval. NA means no ES values.

In several cases, the mean ES values calculated for the functional‐phylogenetic parameter providing the maximum explanation significantly deviated from zero (Figure 3). In spring, the mean SE mostly showed up in the negative range at points K2, K3 and K4. In summer, the mean SE of the sampling points varied around zero with no significant deviations except at point K1, where it was significantly negative. In autumn, the mean SE mostly showed up in the positive range and became significant at points K2 and K4. Index values could not be calculated at points K3 and K5, as here we had empty or single‐species samples, from which no ES value could be derived.

**FIGURE 4:**
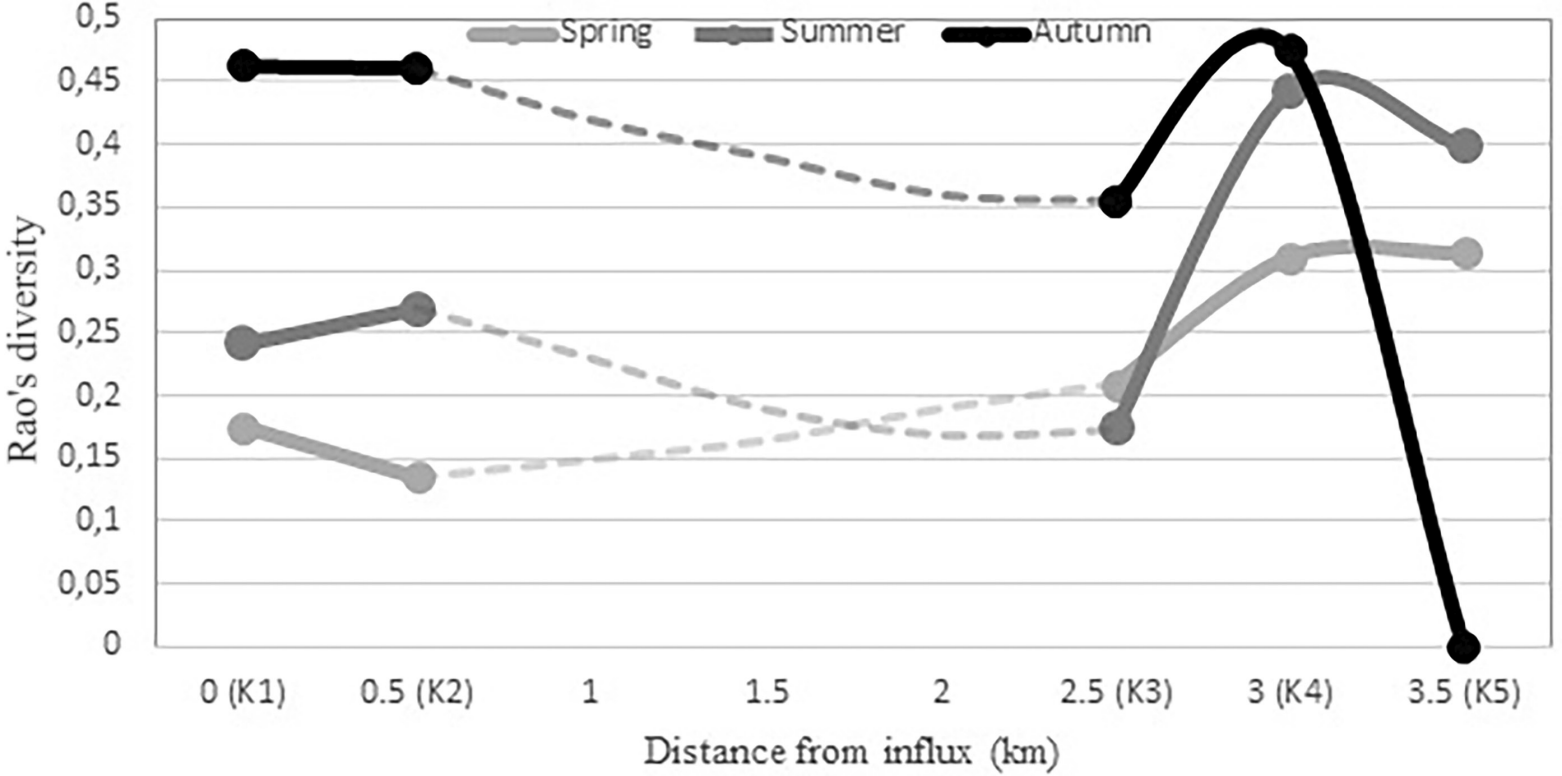
Graph of diversity values calculated with a Rao's quadratic entropy based on the combination of functional and phylogenetic conditions resulting with maximum explanatory power. The values of the points at the examined distance are connected by a dashed line.

In spring, the point farthest from the point of inflow had the highest diversity, while the rotiferan communities were the least diverse at the inflow point (K1) and at its nearest sampling location (K2) (K1) (Figure 4.). In summer, diversity did not depend on the distance from the point of inflow. However, the three points closest to the inflow had lower diversity than the ones farther away. In autumn, the diversity of the farthest (K5) point had clearly the lowest diversity. Point of K4 had the highest diversity, followed by K1, K2 and K3.

## 4 DISCUSSION

In our work, we investigated how nutrient‐rich effluent water from aquaculture influenced the organization of rotiferan communities in a continental oxbow lake. We found that the community organising forces were different in both space (distance from the point of effluence) and time (seasons). The processes that influence community assembly affect not only the number of species but also their ecological similarities and differences (Diaz & Cabido, 2001; McGill et al., 2006). To quantify these, we need to move from species numbers to a functional‐phylogenetic approach (Webb, 2000). Community organization can be guided by three processes. First, a species has to arrive to a potential habitat—so, dispersal limitation is the first ‘filter’. In our study, this was probably not an effective filter: rotiferans have high dispersal power, and the single water body of the oxbow lake posed few dispersal obstacles. Once in loco, abiotic environmental conditions determine whether a species has the appropriate traits to exploit the existing ecological opportunity. This can further be modulated by biotic interactions between community members. We focussed on the combinations of functional ecological and morphological traits of species and their phylogenetic relationships to complement a purely taxonomic assessment (i.e. Tóth et al., 2020). The functional and phylogenetic distances had a significant correlation, showing that the generalization that species belonging to similar functional groups are also phylogenetically closely related (de Bello et al., 2021) also holds for rotiferans. In our case, the functional and phylogenetic distances were not substantially different. Obertegger and Flaim (2015), using five morphological traits (but without a phylogenetic analysis), documented trait convergence in the upper layer of the oligotrophic Lake Tovel in Italy showing that similar traits had repeatedly developed in groups with more distant kinship.

The contribution of the phylogenetic component was the largest in spring, but was still only 25%. In summer and autumn, its influence was much smaller. We found that the ES values were not closely related to the distance from the point of nutrient inflow. However, at points K2–K4, the effect of environmental filtering as a community organizing force was detected. Consequently, the inflow did not create an environmental gradient influencing the organization of rotiferan communities. However, the diversity within 500 m of the point of influence was lower than at points farther away, where the diversity gradually increased with increasing distance. This is consistent with the conclusion from the Rényi diversity curve (Tóth et al., 2020), caused by the high abundance of mesosaprobic species, mainly *Brachionus* spp. Collectively, our results confirm the hypothesis that nutrient subsidy will cause loss of rotiferan diversity, at least in spring.

In summer, community organization was random, except at the point of influx, where the effect of environmental filtering was observed. There was no clear trend by distance from the point of influx. Location K3 had a lower diversity, probably due to the higher nutrient levels measured there (Table S1), which was not detected by evaluating the taxonomic diversity alone (Tóth et al., 2020).

In autumn, the driving force of the community organization shifted towards biotic interactions, although at some points, the number of species and individuals decreased so much that we can no longer speak of a community. During this season, the extra nutrients arriving into the oxbow lake supported the communities rather than reducing their diversity. The claim that nutrient levels and diversity are inversely related does not seem to be evident in all seasons. It may be necessary to investigate the effect of biotic interactions as part of such a functional and phylogenetic analysis.

## 5 CONCLUSION

Considering functional and phylogenetic traits allowed us to identify organising forces of rotifer communities in the largest oxbow lake of the Hungarian Lowland. In spring and summer, environmental filtering played locally important roles in community organization, while biotic interactions were more influential in autumn. Using a combination of functional and phylogenetic diversities, we found that diversity was generally higher farther away from the point of inflow in spring and summer, thus partially supporting the hypothesis that nutrient‐rich conditions reduce functional and phylogenetic diversity in rotifer communities. Oligotrophic conditions allowed higher diversity through reducing the abundance, especially of common species, thus increasing evenness. However, in autumn, the inflow of nutrient‐rich water helped to support the rotiferan community.

The reaction of zooplankton to the impact of aquaculture on aquatic ecosystems through nutrient supply can be better traced by functional and phylogenetic approach than by the traditional taxon‐based diversity analysis. The introduction of functional‐phylogenetic diversity evaluation will allow a more articulate understanding of the reaction of planktonic communities to changes in water quality.

## AUTHOR CONTRIBUTIONS

**Flórián Tóth:** Data curation (lead); formal analysis (lead); investigation (lead); methodology (supporting); project administration (supporting); software (lead); visualization (lead); writing – original draft (lead). **Katalin Zsuga:** Conceptualization (supporting); investigation (supporting); supervision (supporting); writing – review and editing (supporting). **Éva Kerepeczki:** Conceptualization (lead); writing – review and editing (supporting). **Balázs Kovács:** Data curation (supporting); visualization (supporting); writing – review and editing (supporting). **Tibor Magura:** Data curation (supporting); writing – review and editing (supporting). **László Körmöczi:** Methodology (supporting); writing – review and editing (supporting). **Gábor L. Lövei:** Methodology (lead); project administration (lead); supervision (lead); writing – review and editing (lead).

## FUNDING INFORMATION

The experimental work was carried out with the support of the National Agricultural Research and Innovation Centre (now part of the Hungarian University of Agriculture and Life Sciences).

## CONFLICT OF INTEREST STATEMENT

The authors declare that they have no competing financial interests or personal relationships that could have influenced the work reported in this paper.

## Supporting information


Figure S1

## Data Availability

Rotifera abundance data are available via: https://doi.org/10.5061/dryad.c2fqz61fn.
