# Supplementary material for: Discordant spatiotemporal dynamics of functional and phylogenetic diversity of rotiferan communities exposed to aquaculture effluent
Source: Ecol Evol. 2023 Sep 5;13(9):e10503. doi: 10.1002/ece3.10503 (PMC10480067; doi:10.1002/ece3.10503)
Supplement: Supplementary file 1 — Figure S1 [file ECE3-13-e10503-s001.docx]

Supplementary material to the article **“Discordant spatio-temporal dynamics of functional and phylogenetic diversity of rotiferan communities exposed to aquaculture effluent”** by Flórián Tóth^a,b,c,*^, Katalin Zsuga^d^, Éva Kerepeczki^a^, Balázs Kovács^e^, Tibor Magura^f,g^, László Körmöczi^b^ and Gábor L. Lövei^c,e^

^a^Department of Hydrobiology, Research Centre for Aquaculture and Fisheries, Institute of Aquaculture and Environmental Safety, Hungarian University of Agriculture and Life Sciences, 5540 Szarvas, 35 Anna-liget street, Hungary

^b^Department of Ecology, Faculty of Science and Informatics, University of Szeged, 6726 Szeged, 56. Közép fasor, Hungary

^c^Department of Agroecology, Aarhus University, Flakkebjerg Research Center, 4200 Slagelse, Denmark

^d^Agrint Kft, 2100 Gödöllő, 56. Fácán sor, Hungary

^e^Department of Molecular Ecology, Institute of Aquaculture and Environmental Safety, Szent István Campus, Hungarian University of Agriculture and Life Sciences, H-2100 Gödöllő 1 Páter Károly Street, Hungary

^f^ELKH-DE Anthropocene Ecology Research Group, University of Debrecen, H-4010 Debrecen, Egyetem tér 1., Hungary

^g^Department of Ecology, Faculty of Science and Technology, University of Debrecen, H-4032 Debrecen, Egyetem Sq. 1.

^*^Correspondence: Flórián Tóth, Department of Hydrobiology, Research Centre for Aquaculture and Fisheries, Institute of Aquaculture and Environmental Safety, Hungarian University of Agriculture and Life Sciences, H-5540 Szarvas, 35 Anna-liget street, Hungary

Email: toth.florian@uni-mate.hu

**Keywords:** Zooplankton, Biodiversity, Community Ecology, Limnology, Rao Diversity

**Table S1** – Content of Total Nitrogen, Total Phosphorous and Total Suspended Solids of Kákafok oxbow lake by season and sampling location

| **Season** | **Sampling location** | **Total Nitrogen** | **Total Phosphorous** | **Total Suspended Solids** | **Season** | **Sampling location** | **Total Nitrogen** | **Total Phosphorous** | **Total Suspended Solids** | **Season** | **Sampling location** | **Total Nitrogen** | **Total Phosphorous** | **Total Suspended Solids** |
| --- | --- | --- | --- | --- | --- | --- | --- | --- | --- | --- | --- | --- | --- | --- |
|  |  | mg dm^-3^ | mg dm^-3^ | mg/dm^3^ |  |  | mg dm^-3^ | mg dm^-3^ | mg/dm^3^ |  |  | mg dm^-3^ | mg dm^-3^ | mg/dm^3^ |
| Spring1 | K1 | 1.540 | 0.123 | 36.20 | Spring2 | K1 | 2.030 | 0.136 | 31.00 | Spring3 | K1 | 0.909 | 0.117 | 25.40 |
|  | K2 | 1.520 | 0.126 | 36.70 |  | K2 | 1.250 | 0.070 | 27.30 |  | K2 | 0.927 | 0.092 | 13.20 |
|  | K3 | 1.170 | 0.058 | 32.20 |  | K3 | 0.924 | 0.057 | 31.90 |  | K3 | 0.565 | 0.090 | 18.30 |
|  | K4 | 1.010 | 0.054 | 30.30 |  | K4 | 0.927 | 0.060 | 25.60 |  | K4 | 0.277 | 0.041 | 9.70 |
|  | K5 | 1.140 | 0.074 | 30.40 |  | K5 | 0.948 | 0.062 | 20.30 |  | K5 | 0.480 | 0.071 | 8.10 |
| Summer1 | K1 | 4.000 | 0.468 | 39.01 | Summer2 | K1 | 0.517 | 0.073 | 23.70 | Summer3 | K1 | 2.040 | 0.239 | 23.30 |
|  | K2 | 1.930 | 0.260 | 27.20 |  | K2 | 0.927 | 0.101 | 26.70 |  | K2 | 0.712 | 0.113 | 15.20 |
|  | K3 | 2.120 | 0.289 | 54.39 |  | K3 | 0.734 | 0.113 | 20.20 |  | K3 | 0.355 | 0.088 | 12.80 |
|  | K4 | 1.620 | 0.261 | 62.59 |  | K4 | 0.781 | 0.127 | 16.60 |  | K4 | 0.474 | 0.108 | 15.30 |
|  | K5 | 0.924 | 0.210 | 16.20 |  | K5 | 0.698 | 0.124 | 10.00 |  | K5 | 0.520 | 0.114 | 10.00 |
| Autumn1 | K1 | 0.201 | 0.059 | 13.14 | Autumn2 | K1 | 0.195 | 0.053 | 41.10 | Autumn3 | K1 | 1.120 | 0.154 | 18.40 |
|  | K2 | 0.987 | 0.131 | 26.23 |  | K2 | 0.677 | 0.090 | 45.85 |  | K2 | 1.190 | 0.132 | 18.70 |
|  | K3 | 0.103 | 0.094 | 12.40 |  | K3 | 0.218 | 0.069 | 31.40 |  | K3 | 0.391 | 0.070 | 11.50 |
|  | K4 | 0.201 | 0.078 | 16.40 |  | K4 | 0.195 | 0.072 | 30.50 |  | K4 | 0.382 | 0.071 | 7.30 |
|  | K5 | 0.277 | 0.089 | 15.40 |  | K5 | 0.275 | 0.082 | 28.60 |  | K5 | 0.396 | 0.073 | 7.10 |

K1-K5 denote each sampling point away from the point of the introduction of the effluent water of aquaculture

**Table S2** - Ecological and life history traits used to analyse the functional characteristics of Rotifera species

| Species name | Body size (µm) | Trophi type | Feeding type | Physical protection | Body wall type | Corona type | Habitat preference | Tolerance |
| --- | --- | --- | --- | --- | --- | --- | --- | --- |
| *Aplachna priodonta* | 612.4 | incudate | macrophagous omnivores/predators | passive | illoricate | Asplanchna-type | oligo/beta | 5 |
| Bdelloidea sp. | - | ramate | microphagous rotifers | passive | illoricate | Philodina-type | - | - |
| *Brachionus angularis* | 137.5 | malleate | polyphagous rotifers | passive | loricate with spines | Euchlanis/Brachionus-type | beta | 1 |
| *Brachionus calyciflorus* | 273.9 | malleate | polyphagous rotifers | passive | loricate with spines | Euchlanis/Brachionus-type | beta/alfa | 3 |
| *Brachionus diversicornis* | 336.2 | malleate | polyphagous rotifers | passive | loricate with spines | Euchlanis/Brachionus-type | beta | 1 |
| *Brachionus falcatus* | 365.1 | malleate | polyphagous rotifers | passive | loricate with spines | Euchlanis/Brachionus-type | beta | 1 |
| *Brachionus forficula* | 161.2 | malleate | polyphagous rotifers | passive | loricate with spines | Euchlanis/Brachionus-type | beta | 1 |
| *Brachionus leydigi* | 204.9 | malleate | polyphagous rotifers | passive | loricate with spines | Euchlanis/Brachionus-type | beta | 2 |
| *Brachionus nilsoni* | 164.3 | malleate | polyphagous rotifers | passive | loricate with spines | Euchlanis/Brachionus-type | - | - |
| *Brachionus quadridentatus* | 297 | malleate | polyphagous rotifers | passive | loricate with spines | Euchlanis/Brachionus-type | beta | 1 |
| *Brachionus urceolaris* | 213.3 | malleate | polyphagous rotifers | passive | loricate with spines | Euchlanis/Brachionus-type | - | - |
| *Brachionus variabilis* | 337.6 | malleate | polyphagous rotifers | passive | loricate with spines | Euchlanis/Brachionus-type | - | - |
| *Cephalodella biungulata* | 280.6 | virgate | macrophagous algivores | passive | loricate | Notommata-type | oligo/beta | - |
| *Dicranophorus forcipatus* | 331.7 | forcipate | macrophagous omnivores/predators | passive | loricate | Dichranophorus-type | oligo/beta | - |
| *Euchlanis dilatata* | 194.4 | malleate | polyphagous rotifers | passive | loricate | Euchlanis/Brachionus-type | oligo/beta | 3 |
| *Filinia longiseta* | 146.7 | malleoramate | microphagous rotifers | active | illoricate | Conochilus-type | oligo/beta | 3 |
| *Hexarthtra mira* | 253 | malleoramate | microphagous rotifers | active | illoricate | Hexarthra/Testudinella-type | beta | 2 |
| *Keratella quadrata* | 231.6 | malleate | polyphagous rotifers | passive | loricate with spines | Euchlanis/Brachionus-type | oligo/beta | 4 |
| Lepadella sp. | - | malleate | polyphagous rotifers | passive | loricate | Collotheca-type | - | - |
| *Platyias patulus* | 222.5 | malleate | polyphagous rotifers | passive | loricate with spines | Euchlanis/Brachionus-type | beta | 2 |
| *Polyarthra dolycoptera* | 114.2 | virgate | macrophagous algivores | active | illoricate | Euchlanis/Brachionus-type | oligo/beta | - |
| *Synchatea pectinata* | 340 | virgate | macrophagous algivores | passive | illoricate | Asplanchna-type | oligo/beta | 4 |
| *Trichocerca pusilla* | 138.7 | virgate | macrophagous algivores | passive | loricate | Notommata-type | oligo | 2 |
| *Trichotria pocillum* | 200 | malleate | polyphagous rotifers | passive | loricate with spines | Euchlanis/Brachionus-type | oligo | 1 |

Body size - This was characterised by the geometric mean of the size range found in identification keys (Bancsi 1988). Trophi type - The pharyngeal apparatus of rotifers, which is a masticatory apparatus consists of hard, sclerotized, and articulated jaws and muscles organized in specific spatial arrangement, is collectively called trophi (Wallace et al. 2006; Sørensen 2002). Feeding type - The type of food suitable for consumption is determined by the type of trophi and the mode of feeding (Gilbert 2022). Physical protection from predators based on the existence of organs and appendices necessary for movement (Roche 1987). Body wall type – The lorica provide protection against predation (Roche 1987). The presence of caudal spines can be considered phenotypic variability as it can be influenced by the number of predators (Gilbert and McPeek 2013). The animals with less ridged body wall are termed illoricate (Wallace et al. 2006). Corona type - The organ at the apical end of the animal is the corona. This is a ciliated region for food gathering, perception of prey items (Salt 1987) and locomotion (Wallace et al. 2006; Koste 1978) and is linked to mastax and trophi structure (Kutikova 1983). Habitat preference - According to Gulyás (1983), the identified species were classified as beta-, beta/alpha-mesosaprobic, oligo/beta-mesosaprobic or oligosaprobic, according to the status of the waters in which they typically can be found. Tolerance - Based on the occurrence in the various saprobic zones, a tolerance level was also classified (on a scale of 1 (narrow) to 5 (wide)) according to the number of zones the species occurs. In this, 1 values were obtained for the narrow-tolerant species, which were found only in one zone, and 5 for the species occurring in all zones (Gulyás 1983). Species with a frequency of 8:2 or 7:3 in two adjacent sapropic zones received 2 points, as well as those occurring in 3 adjacent zones but at a ratio of 1:8:1. A score of 3 applied to species occurring at a ratio of 6:4 or 5:5 between two adjacent zones or occurred in three saprobic zones but the abundance ratio maximum was 6 or 7 (i.e. 2:6:2 or 2:7:1). The species received a score of 4 if it occurred in three zones but with a maximum ratio of 4-5 (i.e. 3:4:3 or 2:5:2).

**Table S3** - The used cytochrome oxidase subunit 1 sequence access numbers

| Species | Access numbers |
| --- | --- |
| Asplanchna priodonta | MZ438152.1 |
| Bdelloidea sp. | MG449890.1 |
| Brachionus angularis | MZ413879.1 |
| Brachionus calyciflorus | MZ438223.1 |
| Brachionus diversicornis | JN579299.1 |
| Brachionus falcatus | JX216529.1 |
| Brachionus forficula | MZ438218.1 |
| Brachionus leydigi | MZ438219.1 |
| Brachionus plicatilis | AP009408.1 |
| Brachionus quadridentatus | MN627146.1 |
| Brachionus urceolaris | MZ438220.1 |
| Brachionus variabilis | MN427990.1 |
| Cephalodella gibba | AY218089.1 |
| Dicranophorus forcipatus | DQ297771.1 |
| Euchlanis dilatata | HQ944311.1 |
| Filinia longiseta | MN427991.1 |
| Hexarthra mira | MZ438132.1 |
| Keratella quadrata | AF499084.1 |
| Lepadella patella | JX216763.1 |
| Platyias sp. quadricornis | HQ944345.1 |
| Polyarthra dolichoptera | MZ461531.1 |
| Synchaeta pectinata | KP875716.1 |
| Trichocerca capucina | MZ461543.1 |
| Trichotria pocillum | MZ461544.1 |


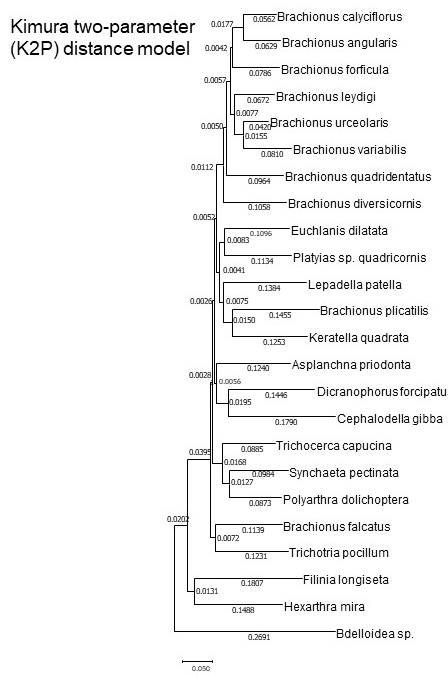


**Figure S1** - Cytochrome Oxidase I haplotypes phylogenetic tree of rotiferan species exposed to aquaculture effluent. The tree was prepared with Neighbour-Joining fitting, taking into account the Kimura-2-parameter model, using a bootstrap value of 3000 with MEGA-X software

**R Script:**

# sample: abundance data (species x sites)

# FDist: functional distance matrix

# PDist: phylogenetic distance matrix

library(picante)

FPDist <- function(PDist, FDist, a = 0.5, p = 2, ord = TRUE){

PDist <- as.matrix(PDist, rownames.force = TRUE)

FDist <- as.matrix(FDist, rownames.force = TRUE)

if(

is.null(rownames(FDist)) ||

is.null(colnames(FDist)) ||

is.null(rownames(PDist)) ||

is.null(colnames(PDist))

) stop('distance matrices must have row and column names')

if(

any(rownames(FDist) != colnames(FDist)) ||

any(rownames(PDist) != colnames(PDist))

) stop('row and column names must match for distance matrices')

if(ord){

FDist <- FDist[order(rownames(FDist)), order(rownames(FDist))]

PDist <- PDist[order(rownames(PDist)), order(rownames(PDist))]

}

if(

any(rownames(FDist) != rownames(PDist)) ||

any(colnames(FDist) != colnames(PDist))

) stop('FDist and PDist must have same row and column names')

FDist <- FDist/max(FDist)

PDist <- PDist/max(PDist)

((a*(PDist^p)) + ((1-a)*(FDist^p)))^(1/p)

}

# i: from 0 to 1 with steps of 0.025

SES <- ses.mpd(sample, FPDist(PDist, FDist, a=i), null.model="sample.pool", abundance.weighted=TRUE, runs=999)

# Gradient: sites codes along the gradient

lm(SES$mpd.obs.z ~ Gradient)

library(boot)

SESmean <- function(SES, d) {

return(mean(SES[d])) }

s1 <- observed mean pairwise distance values for site 1

s2 <- observed mean pairwise distance values for site 2

s3 <- observed mean pairwise distance values for site 3

s4 <- observed mean pairwise distance values for site 4

s5 <- observed mean pairwise distance values for site 5

#si: from s1 to s5

bSES=boot(si, SESmean, R=999)

boot.ci(boot.out = bSES)

#RAO diversity: rot codes data of abundance, FProt codes functional-Phylogenetic distances matrix

library(SYNCSA)

rao.diversity(rot, FProt, phylodist = NULL, checkdata = TRUE,

ord = "metric", put.together = NULL, standardize = TRUE)

#Mantel-test: funcrot codes functional distance matrix, phyrot codes phylogenetic distance matrix

library(ade4)

mantel.rtest(dist(funcrot), dist(phyrot), nrepet = 9999)

**References**

Bancsi, I. (1988). Identification keys for Rotifera II. Vízügyi Hidrobiológia 17. Országos Vízügyi Hivatal, Budapest. [In Hungarian]

Gilbert, J. J. & McPeek, M. A. (2013). Maternal age and spine development in a rotifer: ecological implications and evolution. *Ecology* 94, 2166–2172.

Gilbert, J. J. 2022. Food niches of planktonic rotifers: diversification and implications. *Limnol*. *Oceanogr*. 67, 2218–2251

Gulyás, P. (1983). COMECON Biological methods, *Vízügyi Hidrobiológia* 12, 163-216. [In Hungarian]

Koste, W. (1978). Rotatoria. Die Rädertiere Mitteleuropas, Vol. 2. Gebrüder Borntraeger, Berlin.

Kutikova, L. A. 1983. Parallelism in the evolution of rotifers. *Hydrobiologia* 104, 3–7.

Roche, K. E., (1987). Post-encounter vulnerability of some rotifer prey types to predation by the copepod Acanthocyclops robustus. *Hydrobiologia* 147, 229–233.

Salt, G. W., (1987). The components of feeding behaviour in rotifers. *Hydrobiologia* 147, 271–281.

Sørensen, M. V. (2002). On the evolution and morphology of the rotiferan trophi, with a cladistic analysis of Rotifera. *J. Zool. Syst. Evol. Res*. 40,129–154.

Wallace, R. L., Snell, T. W., Ricci, C., Nogrady, N. (2006) Rotifera biology, ecology and systematics. Backhuys Publishers, Leiden,
